# The antimicrobial peptide nisin promotes host cell survival during SARS-CoV-2 infection

**DOI:** 10.1186/s12985-025-02921-5

**Published:** 2025-10-01

**Authors:** Bevin C. English, Allan Radaic, Ross Barrios-Medina, Kerui Lin, Pachiyappan Kamarajan, Anita Sil, Yvonne L. Kapila

**Affiliations:** 1 CoLabs, University of California San Francisco, San Francisco, CA 94143 USA; 2 Department of Microbiology and Immunology, University of California San Francisco, San Francisco, CA 94143-0414 USA; 3 School of Dentistry, University of California, Los Angeles (UCLA), Los Angeles, CA 90095-1668 USA; 4 Chan Zuckerberg Biohub – San Francisco, San Francisco, CA 94158 USA

**Keywords:** SARS-CoV-2, Nisin, Antimicrobial peptide, Antiviral, Cell death, Bacteriocin

## Abstract

COVID-19 has been a major public health concern for the past five years. While remarkable work has been done to develop therapies, there is still a need for more treatments to fight this disease. Recently, it was suggested that nisin, an FDA-approved antimicrobial compound, may interfere with SARS-CoV-2 entry into host cells. Here, we show that nisin does not inhibit SARS-CoV-2 replication in vitro. Surprisingly, nisin treatment leads to reduced host-cell death during infection in a dose-dependent manner, suggesting that nisin may mitigate SARS-CoV-2-induced pathology.

## Introduction

For the past five years, the world has been battling severe acute respiratory syndrome coronavirus 2 (SARS-CoV-2), the causative agent of coronavirus disease 2019 (COVID-19), which has claimed the lives of over 7 million people [1]. An impressive amount of research has been conducted into COVID-19 prevention and therapeutics, and the clear highlight has been the development of multiple vaccines in a remarkable timeframe [2, 3]. However, these vaccines are not sufficient to eliminate COVID-19 for a number of reasons, and thus there is still a large need for therapies to treat the disease. There are ongoing efforts to target every stage of the viral lifecycle, including viral adsorption and cell entry. Like SARS-CoV-1, SARS-CoV-2 uses angiotensin-converting enzyme 2 (ACE2) as its entry receptor via binding with the viral spike protein [4], and blocking this interaction is a promising therapeutic target [5]. Indeed, many different anti-spike antibodies have been developed as treatments to disrupt this interaction, but a major limitation of this approach is evolution of escape mutants [6]; thus, different strategies are needed to bolster the clinical armamentarium. One key approach for developing therapies for novel diseases is drug repositioning, which has the benefit of investigating compounds with known safety profiles.

Nisin is a lantibiotic antimicrobial peptide (AMP) produced by *Lactococcus lactis*, with variants also produced by other *Streptococcal* species. It acts by inhibiting bacterial cell wall formation and forming pores in the bacterial membrane, leading to bacterial cell death. It was approved by the Joint Food and Agricultural Organization (FAO)/World Health Organization (WHO) as a safe food additive in 1969 and was subsequently approved by the US Food and Drug Administration (USFDA) in 1988 [7]. Extensive work has shown that nisin and its derivatives can inhibit a wide variety of bacterial species in the context of food safety. Intriguingly, nisin has also been shown to have limited antifungal and antiviral properties, though these aspects of nisin are understudied [8]. Further, nisin has immunomodulatory properties, which may complement its direct antimicrobial activities [9]. We have previously demonstrated that nisin significantly reduces the load of periodontal pathogens and attenuates inflammation in both in vitro and in vivo models of periodontal disease, highlighting its therapeutic potential [10]. Nisin has shown efficacy in in vitro and in vivo models of oral cancer, non-alcoholic fatty liver disease (NAFLD), and Alzheimer’s disease-like neuroinflammation, further supporting its broad-spectrum anti-inflammatory and antimicrobial properties [11–15].

Recently, Battacharya et al. performed in silico modeling to show that nisin may bind the human ACE2 receptor with higher affinity than the SARS-CoV-2 spike protein [16]. Thus, we set out to determine if nisin could reduce SARS-CoV-2 infectivity, hypothesizing that nisin would interfere with viral adsorption, thereby reducing viral reproduction.

## Results

### Nisin does not affect Vero cell viability or proliferation

**Fig. 1:**
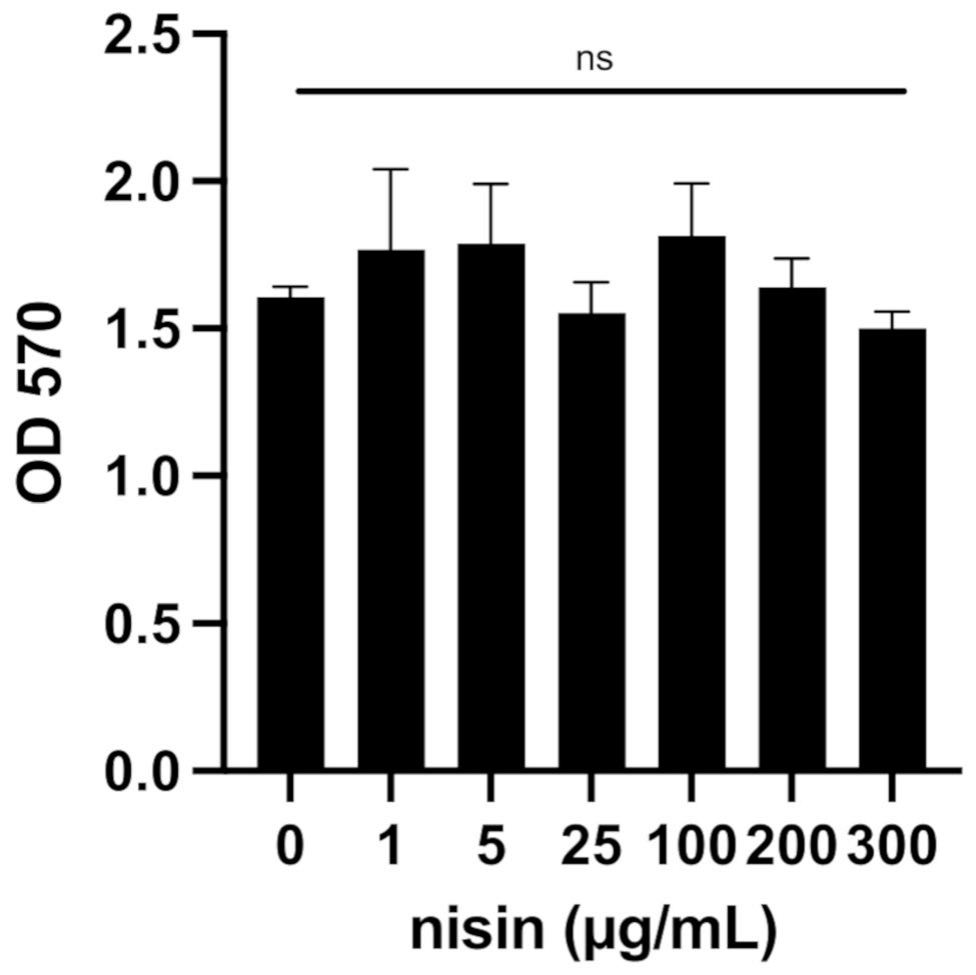
Nisin does not affect Vero E6 viability or proliferation. Vero E6 cells were treated with the indicated concentrations of nisin for 72 h, and cell viability was assessed by MTT assay. ns, not statistically significant, one-way ANOVA

Nisin has been shown to promote cell proliferation or cell death, depending on the cellular context. Nisin reduces the viability of several cell types, including a bovine epithelial cell line, [17], melanoma cells [18], and head and neck squamous cell carcinoma cell lines [19]. However, periodontal ligament cells proliferate after nisin treatment [11]. To assess the impact of nisin on Vero E6 cell viability or proliferation, we measured the metabolic activity of the cells using the MTT assay. After 72 h of treatment, nisin had no effect on Vero cell metabolism at concentrations up to 300 µg/mL (Fig. 1).

### Nisin does not affect peak viral load of SARS-CoV-2

**Fig. 2:**
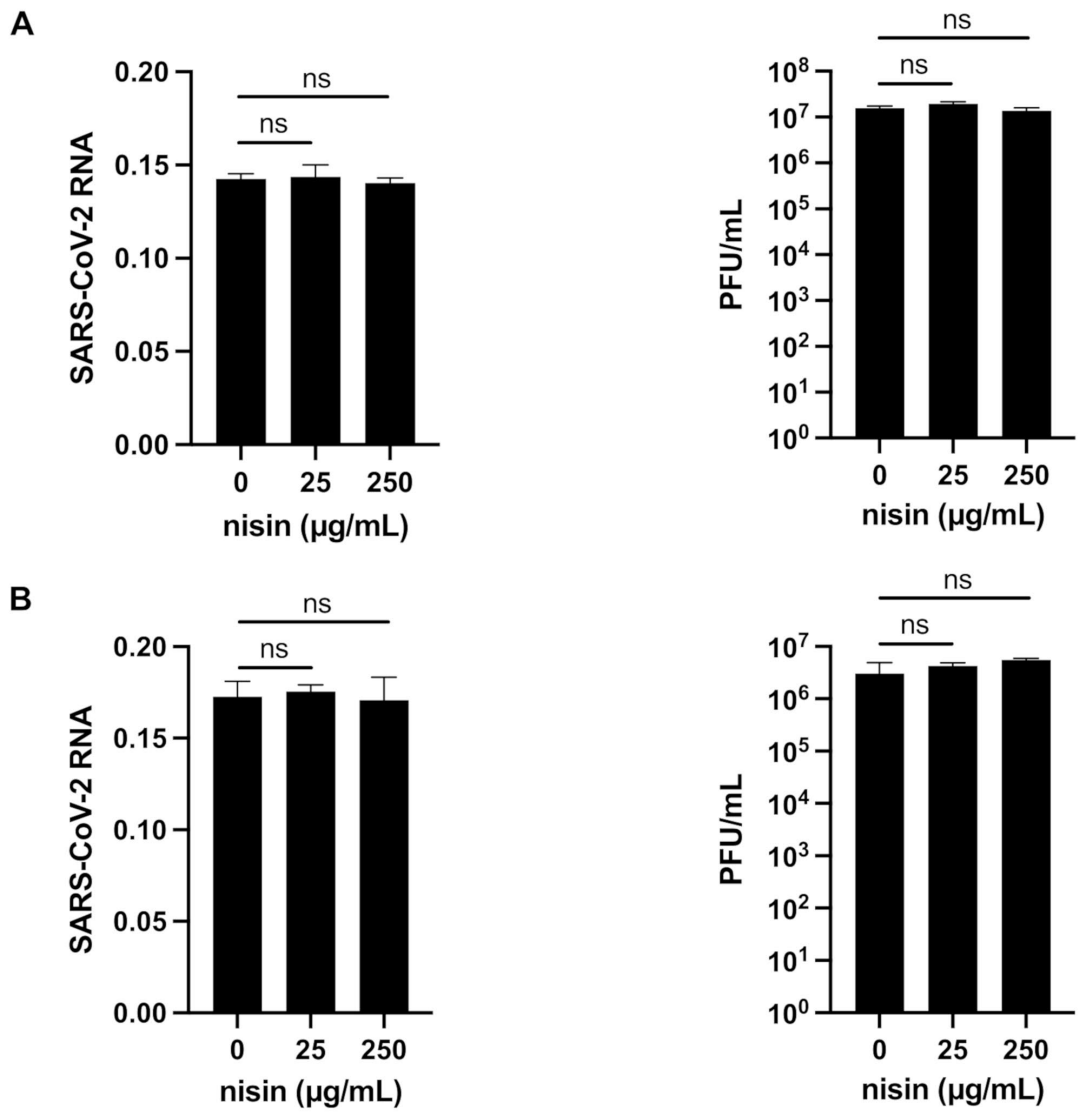
Nisin does not affect peak viral load during infection with SARS-CoV-2. Vero E6 cells were infected with (**A**) the original Wuhan strain (WA1) or (**B**) the delta variant of SARS-CoV-2 and treated with nisin at the indicated concentrations. Viral load was measured by RT-qPCR and plaque forming unit (PFU) enumeration 48 h after infection. “ns” indicates not statistically significant, * indicates *p* < 0.05, ** indicates *p* < 0.01, *** *p* < 0.005, ****p < 0.001, one-way ANOVA with Dunnett’s multiple comparisons test

After confirming that nisin did not affect Vero cell viability, we set out to determine if nisin reduced viral load during SARS-CoV-2 infection. Because we hypothesized that nisin would interfere with viral entry, we pretreated the host cells with nisin and maintained nisin in the culture media throughout infection. Surprisingly, nisin had no effect on viral load of the Wuhan (Fig. 2A) and delta variants (Fig. 2B), as measured by amount of viral genome and infectious viral particles produced.

### Nisin promotes host cell survival during SARS-CoV-2 infection

**Fig. 3:**
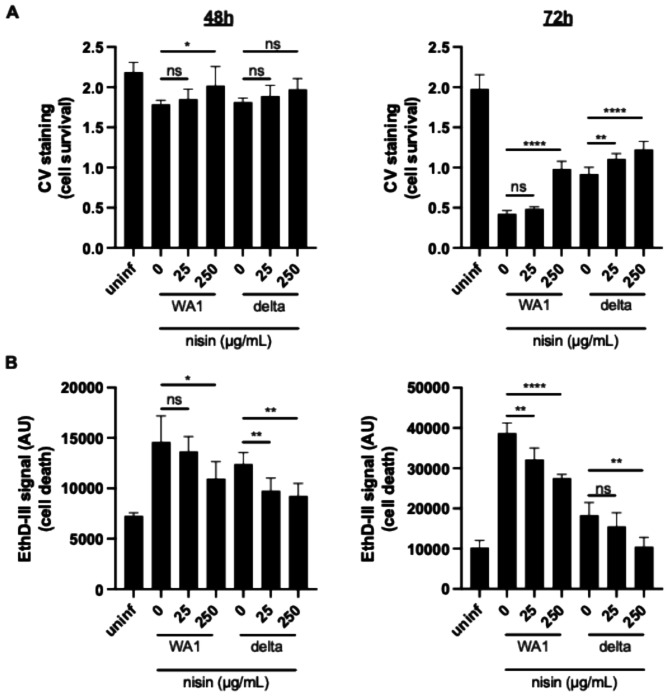
Nisin promotes host cell survival during SARS-CoV-2 infection. Vero E6 cells were infected with the original Wuhan strain (WA1) or the delta variant of SARS-CoV-2 and treated with nisin at the indicated concentrations. Cell viability was measured by crystal violet (CV) staining (**A**) and cell death was measured by ethidium homodimer III (EthD-III) fluorescence (**B**) at 48 and 72 h post-infection. AU, arbitrary units. ns, not statistically significant, * *p* < 0.05, ** *p* < 0.01, *** *p* < 0.005, ****p < 0.001. Samples infected with the same viral strain were analyzed using one-way ANOVA with Dunnett’s multiple comparisons test

After 48 h of SARS-CoV-2 infection, we started to observe cytopathic effects (CPE), most notably cell rounding and loss of the cell monolayer, with severe CPE at 72 h post-infection. Interestingly, we noticed that the nisin-treated cells showed less CPE at both timepoints. To assess the effects of nisin on host-cell viability, we quantified the viable adherent cells using methylene blue. We observed a dose-dependent increase in viable cells during SARS-CoV-2 infection, starting at 48 h post-infection and amplified by 72 h post-infection (Fig. 3A).

This increase in viable cells after nisin treatment could be the result of increased proliferation, a decrease in cell death, or a combination thereof. Because nisin does not increase proliferation of uninfected cells (Fig. 1), we chose to assess cell death using ethidium homodimer III, a nucleic acid dye that stains dead cells with damaged plasma membranes. Nisin treatment caused a dose-dependent decrease in dead cells during infection at 48 and 72 h post-infection with SARS-CoV-2 (Fig. 3B).

## Discussion

We set out to determine if the bacteriocin nisin exhibited antiviral effects on SARS-CoV-2. We hypothesized that nisin would interfere with viral entry into host cells, thereby reducing viral load during infection. Surprisingly, nisin had no effect on peak viral load during in vitro infection. However, we observed that nisin promoted cell survival during SARS-CoV-2 infection in a dose-dependent manner; thus, nisin may ameliorate infection without reducing viral load.

While the antibacterial properties of bacteriocins have been well established, the antiviral properties of these antimicrobial peptides are less well studied and have mixed results. Bacteriocins purified from different bacterial species have been shown to inhibit the enveloped viruses influenza virus A strains H7N7 and H7N1 [20] and herpes simplex virus type 1 (HSV-1) [21]. Additionally, a bacteriocin produced by *Staphylcoccus aureus* inhibits the enveloped Newcastle Disease Virus (NDV) but does not have any effect on the non-enveloped polio virus [22]. It has also been shown that nisin and sakacin A, another bacteriocin, do not have any effect on influenza virus A subtype H1N1, murine norovirus (MNV), NDV, or feline herpesvirus (FHV) [23]; similarly, only very high concentrations of nisin had a slight inhibitory effect on several bacteriophages, leading the authors of the study to conclude that nisin’s antiphage properties were negligible [24]. Nisin has also been shown to cause changes in the oral virome in vivo, but it is unknown if these changes are due to direct effects on viruses, the oral microbiome, or host cells, or some combination thereof [11]. Intriguingly, nisin has been shown to reduce the viral yield and cytopathic effects of bovine viral diarrhea virus (BVDV) infection in vitro [17]. It is unclear exactly how nisin ameliorates BVDV infection: nisin showed no antiviral properties if cells were only pretreated before infection or if nisin was only present during viral adsorption; rather, the greatest reduction in viral load was observed when nisin was present for the entire course of infection [17]. Similarly, we observed that nisin reduced cell death when present for the duration of SARS-CoV-2 infection.

It is unclear exactly how nisin reduces cell death induced by SARS-CoV-2. Nisin has been shown to reduce cytotoxicity in Vero cells after treatment with colistin [25], a potent inducer of apoptosis [26], suggesting that nisin may directly downregulate cell death signaling cascades or may upregulate pro-survival pathways. In addition, nisin treatment has been shown to reduce apoptosis in mesenchymal stem cells after hydrogen peroxide treatment or serum starvation [27, 28]. Intriguingly, there is a concomitant reduction in proinflammatory cytokines and an increase in the anti-inflammatory cytokine IL-10 upon nisin treatment, suggesting that nisin may reduce cell death by altering paracrine or autocrine signaling [27]. Nisin has been shown to modulate cytokine signaling in a variety of experimental models [9], and aberrant cytokine signaling is strongly linked to cell death and severe COVID-19 [29, 30]. An additional mechanism by which nisin may ameliorate SARS-CoV-2 infection is by altering the metabolism of infected cells. SARS-CoV-2 induces metabolic changes in vitro and in vivo, and targeting these changes has been proposed as a potential avenue to develop new therapeutic strategies [31, 32]. Nisin has been shown to alter the metabolism of melanoma cells by reducing their glycolytic capacity [18], but it is unknown how nisin might affect host cell metabolism during SARS-CoV-2 infection. More studies are needed to elucidate how nisin modulates the cellular response to SARS-CoV-2 and promotes host cell survival.

Cell death during viral infection is a double-edged sword for the host; death of infected cells may benefit the host by stopping the production of new virions and remove a replicative niche, but extensive cell death may lead to tissue damage and increased pathology. Cell death is associated with severe COVID-19 [29, 30, 33]. A number of programmed cell death pathways are activated during SARS-CoV-2 infection [34], including apoptosis [35, 36], necroptosis [37, 38], and pyroptosis [39, 40]. Two different inhibitors of apoptosis have been shown to promote host survival and reduce lung damage with modest, if any, effect on viral load in mouse models of SARS-CoV-2 and its close relative Middle East respiratory syndrome coronavirus (MERS-CoV) [41]. Similarly, Li. et al. reported that inhibiting caspase-8 reduces host cell death without affecting SARS-CoV-2 levels in vitro [42]. Additionally, small-molecule inhibition of necroptosis during murine SARS-CoV-2 infection ameliorates lung damage and promotes animal survival without reducing viral load [43]. However, there is some evidence that reduction of host cell death during SARS-CoV-2 infection may not be beneficial in all circumstances: inhibition of pyroptosis in infected human macrophages leads to increased virion production [39]. While more research is needed to better understand the different types of programmed cell death that occur during SARS-CoV-2 infection, targeting host-cell death remains a promising therapeutic avenue for treating COVID-19 [34, 44, 45].

Here, we show that nisin reduces cell death during SARS-CoV-2 infection in vitro in a dose-dependent manner without affecting viral levels. Thus, nisin may be another weapon in the fight against COVID-19, especially when paired with an inhibitor of viral replication, as combination therapy is a promising strategy [46]. Further research is needed to understand the mechanism by which nisin promotes cell survival and how nisin affects SARS-CoV-2 infection in vivo.

## Materials and methods

### Cell culture

Vero E6 and Vero E6/TMPRSS2 cells (kind gifts from Dr. Melanie Ott, UCSF) were cultured in Dulbecco’s Modified Eaglel’s Medium (DMEM; Gibco, USA) supplemented with 10% heat-inactivated fetal bovine serum (FBS; Gibco, USA) and penicillin-streptomycin (Gibco) at 37 °C and 5% CO_2_. An ultra-pure (> 95%) food-grade form of nisin Z (NisinZ^®^ P) was obtained from Handary (Belgium). Nisin stocks (5 mg/mL) were made by dissolving in sterile Milli-Q water protected from light. After constant rotation for 4 h to completely solubilize the nisin powder, nisin stocks were sterile-filtered and stored at 4 °C. The nisin stock was then diluted to the indicated concentrations in serum-free DMEM (Gibco, USA) and added to the cells.

### Nisin titration

Vero E6 cells were seeded in complete media in a 96-well plate at 4 × 10^4^ cells per well. The following day, the supernatant was removed, the cells were washed once with Dulbecco’s PBS (DPBS; Gibso, USA), and serum-free DMEM containing the indicated concentrations of nisin was added to the cells. After 72 h, cell viability was measured using the MTT Cell Proliferation Assay Kit (Cayman Chemical, USA) according to the manufacturer’s instructions, using a BioTek Synergy H1 microplate reader (Agilent, USA) to measure absorbance at 570 nm after 18 h of formazan crystal solubilization.

### SARS-CoV-2 infection

All work with SARS-CoV-2 was conducted at Biosafety Level 3. One day prior to infection, Vero E6 cells were seeded in complete media in 24-well tissue culture plates at 2.5 × 10^5^ cells/well or in black-walled, clear-bottomed 96-well tissue culture plates at 4 × 10^4^ cells/well. The following day, the supernatant was removed, the cells were washed once with DPBS (Gibco, USA), and serum-free DMEM (Gibco, USA) containing the indicated concentrations of nisin was added to the cells. Two hours later, the cells were infected with the Wuhan (USA-WA1/2020 strain) or the delta variant of SARS-CoV-2 (kind gifts from Dr. Melanie Ott, UCSF) at a multiplicity of infection (MOI) of 0.01 in serum-free media containing the indicated concentrations of nisin. After one hour to allow for viral internalization, the cell supernatant was removed and replaced with serum-free DMEM containing the indicated nisin concentrations. A minimum of three wells were infected per condition.

### Plaque assay

Supernatants from infected cells were removed 48 h post-infection and stored at -80 °C until plaque assays were performed as previously described [47]. Briefly, Vero E6/TMPRSS2 + cells, which are commonly used for SARS-CoV-2 plaque assays, were seeded in 12-well tissue culture plates at 5 × 10^5^ cells per well. The following day, ten-fold dilutions of infected cell supernatants were added to the cells for one hour, and then an overlay of 1.2% Avicel RC-581 (International Flavors and Fragrances Inc.) in EMEM (Quality Biological) was added. The cells were incubated at 37 °C and 5% CO_2_ for 72 h. The cells were stained with crystal violet as described below and plaques were enumerated.

### RNA isolation and RT-qPCR

Cells were washed with DPBS and lysed in Buffer RLT (Qiagen, Netherlands). Lysates were stored at -80 °C until RNA was extracted using the RNeasy Mini Kit (Qiagen, Netherlands). Viral RNA was quantified using real-time quantitative polymerase chain reaction (RT-qPCR) using the Luna Universal One-Step RT-qPCR Kit (New England Biolabs, USA), with primers and probes for SARS-CoV-2 proteins N1 (FAM) and N2 (VIC) Research Use Only (RUO; IDT, USA) multiplexed for 45 cycles on QuantStudio 3 Real Time qPCR system (Applied Biosystems, USA) according to the kit’s manual.

### Crystal violet staining

Cells were washed with DPBS and fixed with 4% paraformaldehyde (PFA; Electron Microscopy Sciences, USA). Cells were then stained with a crystal violet solution (20% methanol, 0.1% crystal violet (Fisher Scientific, USA)) and rinsed with water to remove excess stain. To quantify staining levels, crystal violet from dry cells was solubilized in methanol, and absorbance was measured at 570 nm using an Infinite M Nano + plate reader (Tecan, Switzerland).

### Ethidium homodimer III assay

Cells were stained with Ethidium Homodimer III (EthD-III) using the EarlyTox Live/Dead Assay Kit (Molecular Devices, USA) for 1 h according to manufacturer’s instructions. Fluorescence was measured using an Infinite M Nano + plate reader (Tecan, Switzerland).

### Statistical analysis

Data were analyzed with Microsoft Excel (Microsoft) and Prism (GraphPad) using the statistical tests indicated in the figure legends. Data presented here are from a minimum of triplicate measurements from representative experiments.

## Data Availability

No datasets were generated or analysed during the current study.

## Abbreviations

ACE2: Angiotensin converting enzyme 2
ANOVA: Analysis of variance
AU: Arbitrary units
BVDV: Bovine viral diarrhea virus
COVID-19: Coronavirus disease 19
CPE: Cytopathic effects
CV: Crystal violet
DMEM: Dulbecco’s modified Eagle’s medium
DPBS: Dulbecco’s phosphate-buffered saline
EMEM: Eagle’s minimum essential medium
EthD-III: Ethidium homodimer III
FAO: Food and Agricultural Organization
FBS: Fetal bovine serum
FHV: Feline herpes virus
HSV-1: Herpes simplex virus 1
MERS-CoV: Middle East respiratory syndrome coronavirus
MNV: Murine norovirus
MOI: Multiplicity of infection
MTT: 3-(4,5-dimethylthiazolyl-2)-2,5-diphenyltetrazolium bromide
NAFLD: Non-alcoholic fatty liver disease
NDV: Newcastle disease virus
PFA: Paraformaldehyde
PFU: Plaque forming unit
RT-qPCR: Reverse transcription quantitative polymerase chain reaction
RUO: Research use only
SARS-CoV-2: Severe acute respiratory syndrome coronavirus 2
WHO: World Health Organization
